# Facial Feminization Surgery and Quality of Life in Transgender Women: Protocol for a Cohort Study

**DOI:** 10.2196/75065

**Published:** 2025-10-28

**Authors:** Francisca Donoso-Hofer, Rolando Carrasco Soto, Marco Cornejo Ovalle, Conchita Martín

**Affiliations:** 1 Oral and Maxillofacial Surgery Department Faculty of Dentistry University of Chile Santiago Chile; 2 Maxillofacial Service Hospital San Juan de Dios Santiago Chile; 3 Faculty of Dentistry University of Chile Santiago Chile; 4 Faculty of Dentistry Universidad Complutense de Madrid Madrid Spain

**Keywords:** facial feminization surgery, transgender health, quality of life, cephalometry, gender-affirming care, Latin America, psychometric validation

## Abstract

**Background:**

Transgender women face significant health inequities. Facial feminization surgery is an intervention that aligns craniofacial structures with female anatomical norms and gender identity. Although international studies suggest that facial feminization surgery improves psychosocial outcomes, most studies have focused on aesthetic results or relied on generic quality-of-life instruments that have not been validated for this population. In Chile, no study has evaluated the multidimensional impact of facial feminization surgery using patient-centered measures combined with objective cephalometric and photometric analyses. Moreover, no validated Spanish-language quality-of-life instrument exists for transgender women undergoing this surgery.

**Objective:**

This study aims to (1) develop and validate a culturally appropriate quality-of-life questionnaire for transgender women undergoing facial feminization surgery in Chile, and (2) evaluate postoperative changes in quality of life, perception of facial femininity, and cephalometric and photometric parameters.

**Methods:**

The study will include 2 methodological phases: a cross-sectional validation study and a longitudinal cohort study. Phase 1 involves questionnaire development and validation, following the COSMIN (Consensus-Based Standards for the Selection of Health Measurement Instruments) guidelines, with a systematic literature review conducted according to the PRISMA (Preferred Reporting Items for Systematic Reviews and Meta-Analyses) guidelines, expert panel content validation, pilot testing, and psychometric evaluation in a cohort of ≥200 transgender women. Phase 2 is a longitudinal cohort study conducted at San Juan de Dios Hospital in Santiago, Chile, following the STROBE (Strengthening the Reporting of Observational Studies in Epidemiology) guidelines. Thirty transgender women scheduled for facial feminization surgery will be assessed at baseline and 12 months postoperatively. Outcomes include quality of life, self-perceived and third-party–rated femininity, and cephalometric and photometric changes (using cone beam computed tomography). Data analyses include exploratory and confirmatory factor analyses, Cronbach α, paired 1-tailed *t* tests or Wilcoxon signed-rank tests, chi-square or Fisher tests, and linear regression models.

**Results:**

This study was funded in October 2023. Phase 1 (January 2024 to January 2025) included 216 participants, exceeding the minimum sample size requirement. Data analysis is ongoing, and results will be reported separately. Phase 2 is in progress, with preoperative cone beam computed tomography scans and photographs being collected. The validated preoperative questionnaire will be administered in late 2025, with surgeries scheduled throughout 2026. Data collection is expected to conclude in 2027.

**Conclusions:**

This protocol addresses a critical evidence gap in Latin America by integrating psychometric validation and longitudinal evaluation of facial feminization surgery outcomes among transgender women in Chile. The project is expected to develop the first validated Spanish-language quality-of-life instrument specific to facial feminization surgery, alongside multidimensional evidence of surgical outcomes. The findings will inform clinical decision-making, contribute to inclusive patient-centered care, and support the development of evidence-based policies for transgender health in the region.

**International Registered Report Identifier (IRRID):**

DERR1-10.2196/75065

## Introduction

### Background

Some countries face persistent challenges related to health inequities, influenced by factors such as socioeconomic status and geographical distribution. These disparities affect access to health care services across multiple population groups, including transgender individuals.

Transgender women are defined as individuals who identify with the female gender, independent of any medical or surgical interventions they may have undergone [1]. Facial appearance is a key determinant in gender recognition and social perception [2]. Facial characteristics typically associated with masculinity have been linked to increased exposure to social stigma and discrimination, which may contribute to mental health disparities within this population [2-4]. In Chile, available data indicate that the prevalence of depression among transgender individuals is approximately 10 times higher than in the general population, with suicidal ideation rates reaching 68%, compared with 2.2% in the general population [5,6].

Facial feminization surgery is a gender-affirming surgical intervention aimed at modifying facial structures to align with female anatomical norms [7-9]. Although this surgery has been associated with improved psychological and social outcomes, there is limited research evaluating its impact on quality of life using standardized, validated, and population-specific instruments. To date, no studies in Chile have systematically assessed the outcomes of facial feminization surgery, incorporating objective morphological measurements and validated patient-reported outcome measures.

Instruments commonly used to assess quality of life among transgender individuals were originally designed for the general population and later adapted for use in transgender contexts. However, these tools may not comprehensively capture the specific needs and expectations of transgender women undergoing facial feminization surgery [2,3,7,9-12]. Furthermore, most available instruments have been translated from English without undergoing formal validation for use in Spanish-speaking populations, potentially limiting their applicability in Latin American health care settings. Currently, no validated questionnaire exists in Spanish specifically addressing the quality of life of transgender women undergoing facial feminization surgery in Chile [13,14].

The surgery involves modifications to both hard and soft tissues, which can be assessed using cone beam computed tomography (CBCT) and standardized clinical photography. The international literature identifies the frontal-naso-orbital complex (including the forehead, supraorbital crest, frontomalar prominence, temporal ridges, and frontonasal transition) and the mandibular region (chin, mandibular body, angle of the mandible, ascending ramus, and basilar ridge) as the primary anatomical determinants of facial gender. Secondary regions of importance include the nose, zygomatic region, and thyroid cartilage [1,8,15,16]. However, there is a lack of local data in Chile describing the frequency and extent of surgical modifications to these anatomical regions.

Cephalometric differences between male and female facial structures are influenced by biological sex, ethnic background, and genetic inheritance. Typically, male faces have larger craniofacial dimensions, more acute nasolabial angles, and more obtuse mandibular angles, while female face exhibit more convex profiles, smaller nasal dimensions, and less prominent chins [8,15-19].

### Objectives of the Study

This study aims to develop and validate a culturally and linguistically appropriate quality-of-life questionnaire specifically tailored to the transgender population in Chile. It also seeks to evaluate the impact of facial feminization surgery through the analysis of changes in cephalometric and photometric parameters and their association with perceived femininity, as assessed both by third-party evaluators and the participants themselves.

This study hypothesizes that transgender women undergoing facial feminization surgery will experience measurable improvements in quality of life, particularly when facial parameters align more closely with female anatomical standards. In addition, variables such as social participation, employment status, self-perception, and psychosocial stress will be examined to provide a comprehensive understanding of the effects of facial feminization surgery. By integrating multidimensional assessments of psychological well-being, facial morphology, and gender perception, the study aims to generate evidence to inform clinical decision-making and support the development of inclusive, evidence-based health care policies for transgender individuals.

## Methods

### Study Design

This study consists of 2 complementary methodological components: questionnaire development and validation and a longitudinal cohort study.

### Questionnaire Development and Validation

A cross-sectional study will be conducted to develop and psychometrically validate a novel quality-of-life instrument tailored for transgender women undergoing facial feminization surgery in a public hospital in Santiago, Chile. The study will follow the COSMIN (Consensus-Based Standards for the Selection of Health Measurement Instruments) guidelines for the development and evaluation of patient-reported outcome measures (Multimedia Appendix 1). The validation process will include assessments of content validity, structural validity, internal consistency, and reliability in accordance with the COSMIN methodology.

### Longitudinal Cohort Study

A prospective longitudinal cohort analytical study following the STROBE (Strengthening the Reporting of Observational Studies in Epidemiology) guidelines [20] will be conducted to evaluate the effects of facial feminization surgery across three primary domains: (1) quality of life, (2) perception and self-perception of facial femininity, and (3) cephalometric and photometric changes in hard and soft facial tissues (Multimedia Appendix 2).

Quality of life will be assessed using the validated questionnaire, administered preoperatively and at least 12 months postoperatively. Femininity perception will be evaluated through standardized facial photographs assessed by independent raters. Cephalometric and photometric changes will be measured using CBCT and analyzed with Dolphin Imaging software (version 12; Dolphin Imaging and Management Solutions).

### Study Phases

#### Phase 1: Questionnaire Development and Validation

##### Overview

The instrument, titled Quality of Life and Perception of Femininity in Facial Feminization Surgery, will be developed and validated for use in both pre- and postoperative contexts. The development and validation process will follow 5 sequential stages [21,22]. First, a comprehensive review of the scientific literature will be conducted following PRISMA (Preferred Reporting Items for Systematic Reviews and Meta-Analyses) [23] to analyze instruments and tools used to assess quality of life in adult transgender women undergoing facial feminization surgery. The search will be performed in PubMed, Google Scholar, and SciELO databases for studies published between 2010 and 2024 in English or Spanish, using the terms “Quality of life,” “Quality of life instruments,” “Facial feminization surgery,” “Gender affirming surgery,” and “Facial gender affirming surgery” (Multimedia Appendices 3 and 4). Second, the initial construction of the questionnaire will integrate the specific needs and expectations of transgender women undergoing facial feminization surgery, the clinical and technical priorities of the surgical team experienced in facial feminization surgery, and evidence from validated quality-of-life instruments and clinical cases reported by other surgical teams. Third, content validation will be carried out by an expert panel composed of maxillofacial surgeons with experience in facial feminization surgery, sociologists specialized in gender studies, psychologists, public health professionals with expertise in instrument development, and transgender women from civil organizations. Panel members will independently assess the relevance, clarity, language, and cultural appropriateness of each item. A series of meetings will be held between the panel members and the research protocol authors. On the basis of this collaborative process, items will be revised, removed, or added to optimize content validity. Fourth, pilot testing of the preliminary version of the questionnaire will be conducted among transgender women enrolled in the gender identity program at San Juan de Dios Hospital. The objective of this phase will be to evaluate the clarity, acceptability, and comprehensibility of the items, as well as the overall structure and administration time of the instrument. Between 5 and 10 participants will complete the questionnaire in a supervised setting, followed by interviews aimed at identifying any difficulties in item interpretation, relevance of the content, and potential sources of confusion or discomfort. Finally, statistical validation of the questionnaire will involve a larger and more diverse sample of transgender women. Participants will be recruited from multiple sources to ensure variability in sociodemographic backgrounds and health care access. The final version of the questionnaire will be self-administered online.

##### Participants

Participants eligible for the questionnaire validation study must meet the following inclusion criteria: self-identifying as a transgender woman, being aged 18 years or older, living in Chile at the time of questionnaire completion, and being proficient in Spanish (speaking, reading, and comprehension).

##### Recruitment and Questionnaire Distribution

Participants for the validation phase will be recruited through multiple channels to ensure a diverse and representative sample of transgender women in Chile. Recruitment strategies will include telephone outreach to individuals currently enrolled in the gender program at San Juan de Dios Hospital; invitations extended to patients receiving private care from professionals affiliated with the gender program; snowball referrals, whereby participants may suggest other eligible transgender women within their social networks; and open call dissemination through social media platforms and digital networks of civil society organizations.

The questionnaire will be distributed digitally through a hyperlink and hosted on the Google Forms platform. This format will allow participants to complete the instrument remotely at their convenience while ensuring data security and accessibility, and may also help reduce response bias.

##### Sample Size for Questionnaire Validation

Sample size determination for questionnaire validation was based on established methodological recommendations for psychometric studies. While some authors suggest including 5 to 10 participants per item of the instrument, others propose a minimum of 200 participants to ensure sufficient statistical power and stability of factor structures, regardless of the number of items. Following these recommendations, the validation phase of this study will include a minimum of 200 Spanish-speaking transgender women [24,25].

This sample size is deemed sufficient to perform exploratory factor analyses and, when applicable, confirmatory analyses, while also enabling the evaluation of internal consistency and strengthening the generalizability and reliability of the final instrument.

##### Data Analysis

Exploratory and confirmatory factor analyses will be conducted using polychoric correlation matrices and maximum likelihood estimation. Internal consistency will be assessed using Cronbach α, with a threshold of ≥0.70 considered acceptable. All analyses will be independently conducted by 2 researchers to ensure methodological rigor and reproducibility [26].

#### Phase 2: Longitudinal Cohort Study

##### Participants

This cohort will consist of transgender women undergoing facial feminization surgery at the San Juan de Dios Hospital in Santiago, Chile. Participants eligible for the longitudinal cohort study phase must meet the predefined inclusion criteria, as presented in Textbox 1.

**Inclusion criteria.**

- Self-identified transgender women aged ≥18 years
- Currently enrolled in the gender program at San Juan de Dios Hospital
- Clinical indication for facial feminization surgery, including but not limited to genioplasty, mandibular reshaping (angle and basilar crest modification), frontal-nasal-orbital complex remodeling, rhinoplasty, and zygomatic-malar complex remodeling
- Absence of craniomaxillofacial syndromes or contraindications to surgery
- Completion of a multidisciplinary evaluation by a psychologist and a social worker, confirming psychological and emotional readiness for surgery
- Willingness to undergo pre- and postoperative cone beam computed tomography scans and standardized facial photography
- Proficiency in Spanish (speaking, reading, and comprehension)
- Capacity and willingness to provide written informed consent.

##### Sample Size Calculation for Cohort Study

The gender program at San Juan de Dios Hospital currently includes 70 transgender women undergoing hormone therapy and attending regular medical follow-up appointments. On the basis of this population size, a sample of 30 participants was calculated using a 95% confidence level and an estimated margin of error of 12%. This adjustment reflects logistical and ethical considerations related to long-term follow-up while maintaining sufficient statistical validity for exploratory analyses of surgical outcomes.

##### Data Collection

###### Quality of Life Assessment

Participants in the cohort study will complete the validated quality-of-life questionnaire before surgery and again at 12 months postoperatively.

###### Femininity Perception Assessment

Femininity will be assessed through both self-perception and third-party evaluation. Self-perceived femininity will be measured using the questionnaire responses regarding this matter.

To assess changes in third-party perception of femininity, a blind evaluation will be conducted using standardized frontal and lateral clinical photographs taken preoperatively and at least 12 months postoperatively. All images will be coded using an alphanumeric identifier (eg, FFS01, for “facial feminization surgery participant 01”), which will not be visible to evaluators. A control group of cisgender women will be included, using anonymized clinical photographs from the Maxillofacial Surgery Service at San Juan de Dios Hospital, authorized for academic and research purposes.

Forty undergraduate students from nonhealth-related fields (eg, arts, philosophy, architecture, and social sciences) at the University of Chile will serve as evaluators. Participants will be recruited through institutional emails and social media platforms.

Inclusion criteria require students to be aged more than 18 years, without training or experience in health, facial anatomy, or aesthetics. Exclusion applies to students with previous engagement with transgender individuals from the hospital’s gender program. Each student will provide informed consent. Evaluators will rate randomized images (including preoperative, postoperative, and control cases) using a 5-point Likert scale ranging from “feminine appearance” (1) to “non-feminine appearance” (5).

Four groups of 10 evaluators each will be formed, ensuring diversity in gender and age. Each group will be randomly assigned a set of photographs, including preoperative, postoperative, and control images, all displayed in a randomized sequence. As a critical methodological safeguard, pre- and postoperative images of the same patient will not be included within the same group to minimize direct comparative bias.

###### Cephalometric and Photometric Analysis

Facial morphological changes will be measured pre- and postoperatively using CBCT, an imaging software, and extraoral pictures taken in a frontal and lateral view. The following parameters will be assessed: forehead inclination, true vertical distance, nasofrontal angle, mandibular angle, mandibular plane angle, facial profile, nasolabial angle, mentolabial angle, and mentocervical angle.

##### Data Analysis for the Cohort Study

Descriptive statistics will be used to summarize sociodemographic variables (absolute and relative frequencies). The Shapiro-Wilk test will assess the distribution of continuous variables. Normally distributed variables will be reported as mean and SD; nonnormally distributed variables will be reported as median and IQR. Pre- and postoperative comparisons of continuous variables will be made using paired *t* tests or Wilcoxon signed-rank tests, as appropriate. Categorical variables will be compared using Pearson chi-square or Fisher exact test. Linear regression models will evaluate the associations between cephalometric and photometric changes and perceived femininity scores. Statistical significance will be set at *P*≤.05. All analyses will be conducted using Stata (version 11; StataCorp).

### Ethical Considerations

This study will comply with international ethical standards for human participant research, including the principles outlined in the Declaration of Helsinki. To protect participant confidentiality, each participant will be assigned a unique identification code consisting of the acronym FFS (facial feminization surgery) followed by a sequential number based on the order of enrollment (eg, FFS01), which will be used consistently across all phases of the study.

Informed consent will not be required for the questionnaire validation, as this component involves no direct intervention or risk to participants. However, written informed consent will be obtained from all participants enrolled in the longitudinal surgical cohort. Both the research protocol and the informed consent form were approved by the Scientific Ethics Committee of San Juan de Dios Hospital on January 13, 2025. Data will be securely stored in password-protected files and locked in physical storage, accessible only to the authorized research team members. All data will be retained for 5 years following study completion, after which its continued storage will be reassessed; if no longer necessary, it will be permanently destroyed to preserve anonymity.

## Results

This study was funded in October 2023. Phase 1, corresponding to the development and initial psychometric validation of the Quality of Life and Perception of Femininity in Facial Feminization Surgery questionnaire, was conducted between January 2024 and January 2025. A total of 216 transgender women participated in the study, which exceeded the estimated sample size.

The initial questionnaire design was based on a structured literature review conducted according to PRISMA guidelines, which identified existing tools and dimensions used to assess the quality of life of transgender women undergoing facial feminization surgery (Multimedia Appendices 3 and 4). Data analysis is currently in progress, and the corresponding manuscript is expected to be submitted for publication before the end of 2025.

Concurrently, phase 2 of the study—the longitudinal cohort assessing surgical outcomes—is ongoing. Standardized facial photographs and CBCT scans are being collected for presurgical cephalometric and photometric analysis.

The validated presurgical quality-of-life questionnaire will be administered starting in the fourth quarter of 2025 to more than 30 transgender women currently on the waiting list for facial feminization surgery. The surgeries are planned to take place throughout 2026, with the administration of the postsurgical quality-of-life questionnaire starting 12 months after surgery. Data collection is expected to conclude in 2027.

All relevant data underlying the preliminary findings of this study will be made available through the openICPSR repository. Data will be deidentified to protect participant confidentiality in accordance with ethical guidelines. Access to sensitive data that cannot be fully anonymized will be restricted because of ethical considerations. Requests for access to restricted data can be directed to the Scientific Ethics Committee of San Juan de Dios Hospital, Santiago, Chile, and will be considered on a case-by-case basis.

In case of early study termination, an ethically responsible discontinuation plan will be implemented, including notification to the ethics committee and participants, documentation of reasons, appropriate data analysis, and submission of a final report to ensure transparency and scientific value.

## Discussion

### Anticipated Findings

One of the main challenges of this study lies in the complexity of its design. The development and validation of a quality-of-life questionnaire specifically tailored to transgender women in Chile presents a substantial methodological undertaking. The absence of preexisting instruments written and validated in Spanish for this population necessitated the creation of a new tool that is both culturally sensitive and contextually relevant.

The validation process involved multiple stages, including expert review, pilot testing, and large-scale field validation. A total of 216 participants were successfully recruited and completed the questionnaire, exceeding the initial target of 200. This larger sample size enhances the robustness of the psychometric analysis and increases the reliability of the findings. The use of a nonprobabilistic sampling strategy—based on community outreach, institutional networks, and social media—facilitated access to a geographically and socially diverse population but may introduce selection bias, potentially limiting the generalizability of the results to the broader transgender population in Chile.

The longitudinal cohort evaluating surgical outcomes includes 30 participants, a number determined by the eligible population currently enrolled in the gender program at San Juan de Dios Hospital. Although appropriate for exploratory analysis and reflective of real-world clinical constraints, this sample size may reduce the statistical power to detect subtle associations between cephalometric changes and psychosocial variables.

This study has several limitations that should be considered when interpreting the results. First, participant retention during the 12-month follow-up period may be affected by emotional fatigue, life transitions, or reduced interest, potentially leading to attrition and incomplete datasets. Second, the reliance on self-reported measures for quality of life and femininity perception may introduce response bias, particularly in contexts shaped by stigma or social desirability. To minimize this potential bias, the questionnaire was designed to be completed online in a self-administered format, thereby minimizing interviewer influence and reducing the likelihood of socially desirable responding. Third, while third-party assessments are included to complement self-perceptions, evaluations of gender expression and facial aesthetics remain inherently subjective and culturally variable. Finally, although a control group of cisgender women is incorporated, normative comparisons may still influence the interpretation of femininity scores.

Despite these challenges, this study is supported by several strengths that enhance its feasibility and scientific relevance. The project is developed in a health network that serves a significant proportion of Santiago’s population, ensuring access to clinical infrastructure, a well-defined study population, and robust ethical oversight. Collaboration with civil society organizations in Santiago strengthens recruitment and fosters meaningful community engagement. To mitigate attrition risks, this study will implement participant-centered retention strategies, including regular follow-up through mobile messaging and social media to address potential geographic and logistical barriers.

Ultimately, this study is expected to make a valuable contribution to transgender health research in Latin America by addressing the critical evidence gap and introducing a validated, culturally appropriate instrument for clinical and academic use.

### Conclusions

This study protocol outlines a 2-phase research project designed to address critical gaps in the evaluation of facial feminization surgery among transgender women in Chile. By developing and validating a culturally appropriate quality-of-life questionnaire and by assessing postoperative changes in femininity perception and facial morphology, this study aims to provide multidimensional evidence to inform both clinical practice and public health strategies. Despite logistical and methodological challenges, the integration of quantitative, anatomical, and psychosocial data is expected to generate meaningful insights that will contribute to more inclusive, patient-centered gender-affirming care in Latin America.

## Appendix

Multimedia Appendix 1 COSMIN-based checklist for reporting the development and validation of a patient-reported outcome measure.

Multimedia Appendix 2 STROBE statement—checklist of items that should be included in reports of observational studies.

Multimedia Appendix 3 Study characteristics and quality of life assessment.

Multimedia Appendix 4 PRISMA flow diagram for inclusion and exclusion of studies.

Multimedia Appendix 5 Peer review report from the Research Directorate (DIFO) of the Faculty of Dentistry, University of Chile.

## Abbreviations

CBCT: cone beam computed tomography
COSMIN: Consensus-Based Standards for the Selection of Health Measurement Instruments
PRISMA: Preferred Reporting Items for Systematic Reviews and Meta-Analyses
STROBE: Strengthening the Reporting of Observational Studies in Epidemiology

## References

[1] Coleman E, Radix AE, Bouman WP, Brown GR, de Vries AL, Deutsch MB, Ettner R, Fraser L, Goodman M, Green J, Hancock AB, Johnson TW, Karasic DH, Knudson GA, Leibowitz SF, Meyer-Bahlburg HF, Monstrey SJ, Motmans J, Nahata L, Nieder TO, Reisner SL, Richards C, Schechter LS, Tangpricha V, Tishelman A, Van Trotsenburg MA, Winter S, Ducheny K, Adams NJ, Adrián TM, Allen LR, Azul D, Bagga H, Başar K, Bathory DS, Belinky JJ, Berg D, Berli JU, Bluebond-Langner RO, Bouman M, Bowers ML, Brassard PJ, Byrne J, Capitán L, Cargill CJ, Carswell JM, Chang SC, Chelvakumar G, Corneil T, Dalke KB, De Cuypere G, de Vries E, Den Heijer M, Devor AH, Dhejne C, D'Marco A, Edmiston EK, Edwards-Leeper L, Ehrbar R, Ehrensaft D, Eisfeld J, Elaut E, Erickson-Schroth L, Feldman JL, Fisher AD, Garcia MM, Gijs L, Green SE, Hall BP, Hardy TL, Irwig MS, Jacobs LA, Janssen AC, Johnson K, Klink DT, Kreukels BP, Kuper LE, Kvach EJ, Malouf MA, Massey R, Mazur T, McLachlan C, Morrison SD, Mosser SW, Neira PM, Nygren U, Oates JM, Obedin-Maliver J, Pagkalos G, Patton J, Phanuphak N, Rachlin K, Reed T, Rider GN, Ristori J, Robbins-Cherry S, Roberts SA, Rodriguez-Wallberg KA, Rosenthal SM, Sabir K, Safer JD, Scheim AI, Seal LJ, Sehoole TJ, Spencer K, St Amand C, Steensma TD, Strang JF, Taylor G, Tilleman K, T'Sjoen GG, Vala LN, Van Mello NM, Veale JF, Vencill J, Vincent B, Wesp LM, West MA, Arcelus J (2022). Standards of care for the health of transgender and gender diverse people, version 8. Int J Transgend Health.

[2] Morrison SD, Capitán-Cañadas F, Sánchez-García A, Ludwig D, Massie J, Nolan I, Swanson M, Rodríguez-Conesa M, Friedrich J, Cederna P, Bellinga R, Simon DC, Capitán L, Satterwhite T (2020). Prospective quality-of-life outcomes after facial feminization surgery: an international multicenter study. Plast Reconstr Surg.

[3] Bockting W, Coleman E, Deutsch MB, Guillamon A, Meyer I, Meyer W, Reisner S, Sevelius J, Ettner R (2016). Adult development and quality of life of transgender and gender nonconforming people. Curr Opin Endocrinol Diabetes Obes.

[4] T'Sjoen G, Arcelus J, Gooren L, Klink D, Tangpricha V (2019). Endocrinology of transgender medicine. Endocr Rev.

[5] Kohnepoushi P, Nikouei M, Cheraghi M, Hasanabadi P, Rahmani H, Moradi M, Moradi G, Moradpour F, Moradi Y (2023). Prevalence of suicidal thoughts and attempts in the transgender population of the world: a systematic review and meta-analysis. Ann Gen Psychiatry.

[6] Guzmán-González M, Barrientos J, Saiz JL, Gómez F, Cárdenas M, Espinoza-Tapia R, Bahamondes J, Lovera L, Giami A (2020). Salud mental en población transgénero y género no conforme en Chile. Rev Med Chil.

[7] Ainsworth TA, Spiegel JH (2010). Quality of life of individuals with and without facial feminization surgery or gender reassignment surgery. Qual Life Res.

[8] Capitán L, Gutiérrez Santamaría J, Simon D, Coon D, Bailón C, Bellinga R, Tenório T, Capitán-Cañadas F (2020). Facial gender confirmation surgery: a protocol for diagnosis, surgical planning, and postoperative management. Plast Reconstr Surg.

[9] Chou DW, Bruss D, Tejani N, Brandstetter K, Kleinberger A, Shih C (2022). Quality of life outcomes after facial feminization surgery. Facial Plast Surg Aesthet Med.

[10] Alper DP, Almeida MN, Hu KG, De Baun HM, Hosseini H, Williams MC, Salib A, Shah JA, Persing J, Alperovich M (2023). Quantifying facial feminization surgery's impact: focus on patient facial satisfaction. Plast Reconstr Surg Glob Open.

[11] Nobili A, Glazebrook C, Arcelus J (2018). Quality of life of treatment-seeking transgender adults: a systematic review and meta-analysis. Rev Endocr Metab Disord.

[12] Rosales O, Sejdiu Z, Camacho JM, Quindlen CE, Herr SJ, Yasback A, Patel H, Sharma D, Brandt K, Behnam A (2023). Facial feminization procedures and its impact on quality of life: a mini review. Health Sci Rev.

[13] Morrison SD, Crowe CS, Wilson SC (2017). Consistent quality of life outcome measures are needed for facial feminization surgery. J Craniofac Surg.

[14] Raner GA, Jaszkul KM, Bonapace-Potvin M, Al-Ghanim K, Bouhadana G, Roy A, Bensimon ? (2024). Quality of life outcomes in patients undergoing facial gender affirming surgery: a systematic review and meta-analysis. Int J Transgend Health.

[15] Lee JC, Pfaff MJ, Lee JC (2020). Discussion: prospective quality-of-life outcomes after facial feminization surgery: an international multicenter study. Plast Reconstr Surg.

[16] Ramly EP, Katave C, Ranganathan K (2024). Facial feminization: upper third of the face. Oral Maxillofac Surg Clin North Am.

[17] Telang PS (2020). Facial feminization surgery: a review of 220 consecutive patients. Indian J Plast Surg.

[18] Arnett GW, Jelic JS, Kim J, Cummings DR, Beress A, Worley C, Chung B, Bergman R (1999). Soft tissue cephalometric analysis: diagnosis and treatment planning of dentofacial deformity. Am J Orthod Dentofacial Orthop.

[19] Ihnat JM, Hu KG, Parikh N, Almeida MN, Williams M, Hauc SC, Alperovich M (2025). Quantification of cephalometric changes in gonial angle morphology following facial feminization surgery. J Craniofac Surg.

[20] Cuschieri S (2019). The STROBE guidelines. Saudi J Anaesth.

[21] Johnson C, Aaronson N, Blazeby JM, Bottomley A, Fayers P, Koller M, Kuliś D, Ramage J, Sprangers M, Velikova G, Young T, EORTC Quality of Life Group (2011). Guidelines for developing questionnaire modules. European Organisation for Research and Treatment of Cancer.

[22] Feil K, Riedl D, Gschwentner L, Vomstein K, Wegscheider J, Arnold E, Toth B (2022). Development of a quality of life questionnaire for transgender individuals during hormone therapy (iTransQol). Arch Gynecol Obstet.

[23] Page MJ, McKenzie JE, Bossuyt PM, Boutron I, Hoffmann TC, Mulrow CD, Shamseer L, Tetzlaff JM, Akl EA, Brennan SE, Chou R, Glanville J, Grimshaw JM, Hróbjartsson A, Lalu MM, Li T, Loder EW, Mayo-Wilson E, McDonald S, McGuinness LA, Stewart LA, Thomas J, Tricco AC, Welch VA, Whiting P, Moher D (2021). The PRISMA 2020 statement: an updated guideline for reporting systematic reviews. BMJ.

[24] Roco Videla Á, Hernández Orellana M, Silva González O (2021). What is the appropriate sample size to validate a questionnaire?. Nutr Hosp.

[25] Espinoza I, Osorio P, Torrejón MJ, Lucas-Carrasco R, Bunout D (2011). Validación del cuestionario de calidad de vida (WHOQOL-BREF) en adultos mayores chilenos. Rev Méd Chile.

[26] Heo M, Kim N, Faith MS (2015). Statistical power as a function of Cronbach alpha of instrument questionnaire items. BMC Med Res Methodol.

