# Supplementary material for: Facial Feminization Surgery and Quality of Life in Transgender Women: Protocol for a Cohort Study
Source: JMIR Res Protoc. 2025 Oct 28;14:e75065. doi: 10.2196/75065 (PMC12605289; doi:10.2196/75065)
Supplement: Multimedia Appendix 1 [file resprot_v14i1e75065_app1.docx]

Multimedia Appendix 1: COSMIN-based checklist for reporting the development and validation of a patient-reported outcome measure (PROM)

**Facial Feminization Surgery and Quality of Life in Transgender Women: Protocol for a Cohort Study on Morphological and Psychosocial Outcomes**

| Domain | Item | Evaluation | Comments/Justification |
| --- | --- | --- | --- |
| I. General recommendation | (1) Research aim  (2) Construct description  (3) Development process  (4) Conceptual model  (5) PROM structure & scoring  (6) Existing evidence  (7) Context of use  (8) Inclusion/exclusion criteria  (9) Sampling method  (10) Representativeness | Very Good  Very Good  Very Good  Very Good  Very Good  Very Good  Very Good  Very Good  Very Good  Adequate | Clearly stated in the introduction and objectives. A PROM for transgender women undergoing facial feminization surgery.  The construct "quality of life and perception of femininity" is explicitly described.  Described in detail: literature review, expert panel, pilot testing, statistical validation.  Reflective model is implied; psychometric methods suggest such assumption.  The manuscript mentions item evaluation and factor analysis. Detailed scoring algorithm is not yet presented.  Literature review identifies gaps in existing tools; references support this.  Clearly defined for clinical and research use in Spanish-speaking trans women undergoing facial feminization surgery.  Thoroughly described.  Described: multi-source recruitment (hospital program, snowballing, social media).  Diverse sources mentioned, but limitations acknowledged in Discussion. |
| II. Content Validity | (1) Appropriate method from patient perspective | Very good | Relevance, clarity, and comprehensibility assessed in pilot phase with 5–10 participants and follow-up interviews. |
|  | (2) Appropriate method from professional perspective | Very good | Appropriate qualitative methods were used, including independent expert review and consensus meetings to evaluate item relevance and overall comprehensiveness. |
|  | (3) Inclusion of professionals from all relevant disciplines | Very good | Panel included surgeons, sociologists, public health experts, and trans women. |
|  | (4) Adequate number of participants (≥7 qualitative, ≥50 quantitative) | Very good | 5–10 participants for pilot; more than 200 in statistical validation. |
|  | (5) Use of topic guide and transcription of interviews | Adequate | An open feedback approach was used without a structured topic guide; comments were noted during group discussions, but interviews were not transcribed verbatim. |
|  | (6) Two or more researchers involved in analysis | Very good | Content validity data will be analysed by two researchers to ensure rigor and reduce bias |
|  | (7) Appropriate qualitative/quantitative analysis method | Very good | A thematic analysis will be conducted on qualitative feedback to identify issues of clarity, relevance, and comprehensiveness across items. |
| II. Structural Validity | (8) Reflective theoretical model defined  (9) Confirmatory factor analysis performed  (10) Adequate sample size for analysis (e.g., 7×items + ≥100 for CFA) | Very good  Very good  Very good | The questionnaire is based on a reflective model, if all items represent manifestations of the same underlying construct.  Exploratory and confirmatory factor analysis planned.  >200 participants; meets COSMIN standards for factor analysis. |
| III. Internal Consistency | (11) Unidimensionality established  (12) Cronbach’s alpha or Omega calculated | Adequate  Very good | Assumed through factor analysis; not detailed per subscale.  Planned with ≥0.70 threshold |
| IV. Reliability | (13) ICC, Kappa or agreement coefficient calculated  (14) Similar test conditions between measurements | Not applicable  Very good | Measurements will be conducted under similar conditions regarding setting, instructions, and mode of administration. |
| V. Criterion Validity | (15) Existence and justification of 'gold standard'  (16) Correlations or AUC calculated | Not applicable  Very good | Correlations between pre- and post-surgery scores will be calculated to assess responsiveness. |
| VI. Construct Validity | (17) Clear hypotheses defined before testing  (18) Psychometric properties of comparator clearly described | Adequate  Not applicable | A priori hypotheses were formulated, anticipating improvements in quality of life and psychosocial outcomes. |
| VII. Responsiveness | (19) Expected change hypotheses clearly stated  (20) Appropriate interval between measurements | Not applicable  Very good | No detailed hypotheses about expected score changes yet. Ongoing data.  An interval of 12 months between pre- and post-surgery measurements was selected to capture stable postoperative outcomes. |
| VIII. Translation Process | (21) Forward and backward translation with appropriate linguistic standards  (22) Pilot testing performed in target population | Not applicable  Very good | Translation was not required, as the questionnaire was originally developed in Spanish for use in a Spanish-speaking population.  Pilot testing was conducted with transgender women from the target population to assess clarity, relevance, and acceptability of the items. |
