# Supplementary material for: Facial Feminization Surgery and Quality of Life in Transgender Women: Protocol for a Cohort Study
Source: JMIR Res Protoc. 2025 Oct 28;14:e75065. doi: 10.2196/75065 (PMC12605289; doi:10.2196/75065)
Supplement: Multimedia Appendix 2 [file resprot_v14i1e75065_app2.docx]

Multimedia Appendix 2: STROBE Statement—checklist of items that should be included in reports of observational studies

**Facial Feminization Surgery and Quality of Life in Transgender Women: Protocol for a Cohort Study on Morphological and Psychosocial Outcomes**

|  | Item No. | Recommendation | Page  No. | Relevant text from manuscript |
| --- | --- | --- | --- | --- |
| **Title and abstract** | 1 | (*a*) Indicate the study’s design with a commonly used term in the title or the abstract | 1- metadata | The study design is indicated in both the title ("Protocol for a Cohort Study") and abstract using a standard term. |
|  |  | (*b*) Provide in the abstract an informative and balanced summary of what was done and what was found | Metadata | The abstract provides a clear and informative summary of the study’s rationale, objectives, methodology, current progress, and expected outcomes. |
| Introduction | | | |  |
| Background/rationale | 2 | Explain the scientific background and rationale for the investigation being reported | 1-2 | The introduction outlines the scientific rationale, emphasizing health disparities affecting transgender women in Chile and the psychological relevance of facial appearance. It highlights the lack of validated quality-of-life instruments in Spanish for this population and the need for culturally relevant evaluation of facial feminization surgery outcomes. |
| Objectives | 3 | State specific objectives, including any prespecified hypotheses | 3 | The study aims to develop and validate a culturally and linguistically appropriate quality-of-life questionnaire for transgender women in Chile, and to evaluate the impact of facial feminization surgery through cephalometric and photometric changes and their association with perceived femininity. The hypothesis is explicitly stated. |
| Methods | | | |  |
| Study design | 4 | Present key elements of study design early in the paper | 3-4 | The manuscript describes a two-phase design: (1) cross-sectional validation study for questionnaire development, and (2) longitudinal cohort study to assess surgical outcomes. These elements are presented early in the “Methods” section. |
| Setting | 5 | Describe the setting, locations, and relevant dates, including periods of recruitment, exposure, follow-up, and data collection | 7-10 | The study is conducted at San Juan de Dios Hospital in Santiago, Chile. Phase 1 took place between January 2024 and January 2025; Phase 2 is ongoing, with surgeries planned throughout 2026 and follow-up until 2027. |
| Participants | 6 | (*a*) *Cohort study*—Give the eligibility criteria, and the sources and methods of selection of participants. Describe methods of follow-up  *Cross-sectional study*—Give the eligibility criteria, and the sources and methods of selection of participants | 6-7 | Eligibility criteria are clearly detailed for both phases. For the validation study: self-identified transgender women aged ≥18, Spanish proficiency, and Chilean residency. For the surgical cohort: enrolled in the gender program, indication for surgery, psychological readiness, and consent to imaging. Methods of recruitment and follow-up are described. |
|  |  |  |  |  |
| Variables | 7 | Clearly define all outcomes, exposures, predictors, potential confounders, and effect modifiers. Give diagnostic criteria, if applicable | 3-4 | Primary outcomes include quality of life, femininity perception, and morphological changes. Predictors such as social participation and stress are included. No effect modifiers or diagnostic criteria reported. |
| Data sources/ measurement | 8* | For each variable of interest, give sources of data and details of methods of assessment (measurement). Describe comparability of assessment methods if there is more than one group | 4 | Quality of life assessed via validated questionnaire; femininity via self-report and third-party ratings using standardized photos; facial morphology via CBCT and photo analysis. |
| Bias | 9 | Describe any efforts to address potential sources of bias | 11-12 | Bias mitigated through blinded photo assessments, randomized image sequence, exclusion of evaluators with medical background, and separation of pre/post-op images. |
| Study size | 10 | Explain how the study size was arrived at | 6-7 | Sample size: 216 for questionnaire validation (target: 200); 30 for cohort study based on 70 candidates with 95% CI and ±12% margin. |

| Quantitative variables | | 11 | | Explain how quantitative variables were handled in the analyses. If applicable, describe which groupings were chosen and why | 6 | | Factor analysis and Cronbach’s alpha (≥0.70) for reliability; paired t-tests or Wilcoxon for pre/post comparisons. |
| --- | --- | --- | --- | --- | --- | --- | --- |
| Statistical methods | | 12 | | (*a*) Describe all statistical methods, including those used to control for confounding | 6-9 | | Statistical tests include paired t/Wilcoxon, chi-square/Fisher, and linear regression. |
|  |  |  |  | (*b*) Describe any methods used to examine subgroups and interactions |  | | No subgroups and interactions data reported. Study ongoing. |
|  |  |  |  | (*c*) Explain how missing data were addressed |  | | No missing data reported. Study ongoing. |
|  |  |  |  | (*d*) *Cohort study*—If applicable, explain how loss to follow-up was addressed  *Cross-sectional study*—If applicable, describe analytical methods taking account of sampling strategy | 4-5 | | A non-probabilistic sampling strategy was used. Analyses included exploratory and confirmatory factor analysis using polychoric correlations, and internal consistency was assessed via Cronbach’s alpha. |
| Results | | | | | | | |
| Participants | | 13* | | (a) Report numbers of individuals at each stage of study—eg numbers potentially eligible, examined for eligibility, confirmed eligible, included in the study, completing follow-up, and analysed | 10 | | 216 participants completed questionnaire; 30 in surgical cohort that will start 2026 |
|  |  |  |  | (b) Give reasons for non-participation at each stage |  | | Non-participation data reported yet. Study ongoing. |
|  |  |  |  | (c) Consider use of a flow diagram |  | | Will do when study is finished |
| Descriptive data | | 14* | | (a) Give characteristics of study participants (eg demographic, clinical, social) and information on exposures and potential confounders |  | | No participant characteristics reported yet since this an ongoing study. |
|  |  |  |  | (b) Indicate number of participants with missing data for each variable of interest |  | | No missing data reported. Study ongoing. |
|  |  |  |  | (c) *Cohort study*—Summarise follow-up time (eg, average and total amount) | 4 | | The cohort study includes a planned follow-up period of 12 months for each participant after surgery. |
| Outcome data | | 15* | | *Cohort study*—Report numbers of outcome events or summary measures over time |  | | Outcome data not reported; data analysis ongoing |
|  |  |  |  | *Cross-sectional study—*Report numbers of outcome events or summary measures |  | | Outcome data not reported; data analysis ongoing |
| Main results | | 16 | | (*a*) Give unadjusted estimates and, if applicable, confounder-adjusted estimates and their precision (eg, 95% confidence interval). Make clear which confounders were adjusted for and why they were included |  | | No unadjusted/adjusted estimates or confidence intervals yet; manuscript preparation pending. |
|  |  |  |  | (*b*) Report category boundaries when continuous variables were categorized |  | | Data analysis ongoing |
|  |  |  |  | (*c*) If relevant, consider translating estimates of relative risk into absolute risk for a meaningful time period |  | | Not applicable. This study does not involve risk comparisons between exposed and unexposed groups. |
| Other analyses | 17 | | Report other analyses done—eg analyses of subgroups and interactions, and sensitivity analyses | |  | No subgroup or sensitivity analyses reported; study and data analysis ongoing | |
| Discussion | | | | | | | |
| Key results | 18 | | Summarise key results with reference to study objectives | | 10 | Key result: 216 recruited participants exceed the original target, improving psychometric robustness. Study and data analysis ongoing | |
| Limitations | 19 | | Discuss limitations of the study, taking into account sources of potential bias or imprecision. Discuss both direction and magnitude of any potential bias | | 10-11 | Acknowledged: follow-up loss, response bias, subjective aesthetic evaluations, and normative bias from control group. | |
| Interpretation | 20 | | Give a cautious overall interpretation of results considering objectives, limitations, multiplicity of analyses, results from similar studies, and other relevant evidence | |  | No overall interpretation of results reported. Study ongoing. | |
| Generalisability | 21 | | Discuss the generalisability (external validity) of the study results | | 10-11 | Non-probabilistic sample may limit broader applicability to Chilean transgender population. | |
| Other information | | |  | | | | |
| Funding | 22 | | Give the source of funding and the role of the funders for the present study and, if applicable, for the original study on which the present article is based | |  | The publication of the study is funded by the University of Chile's Faculty of Dentistry. Funders had no role in the study design or reporting. | |
