# Supplementary material for: Facial Feminization Surgery and Quality of Life in Transgender Women: Protocol for a Cohort Study
Source: JMIR Res Protoc. 2025 Oct 28;14:e75065. doi: 10.2196/75065 (PMC12605289; doi:10.2196/75065)
Supplement: Multimedia Appendix 3 [file resprot_v14i1e75065_app3.docx]

Multimedia Appendix 3: Study characteristics & Quality of life assessment

| **Study** | Location | Journal | Type of study | Quality of life assessment tool | QoL Dimension |
| --- | --- | --- | --- | --- | --- |
| Chou et al.,2022 USA Facial Plastic Surgery & Aesthetic Medicine Case series (n=107) | | | | Pre and post op outcome evaluation (9 questions) | Facial appearance, femineity perception, social and work life |
| Morrison et al.,2020 | USA & Spain | Plastic and Reconstructive Surgery | Cohort (n=66) | Pre and post op survey adapted from general facial aesthetic surgery assessment tool | Physical, emotional, and social domains of patient satisfaction with their face. |
| Caprini et al.,2023 | USA | Annals of Surgery | Observational Cross-Sectional Comparative study (n=169) | Pre and post op scores from 11 validated PROMIS instruments | Various domains of mental, physical, and social health, including anxiety, depression, anger, overall well-being, sexual satisfaction, and social connection. |
| Ainsworth & Spiegel | USA | Quality of Life Research | Observational Cross-Sectional Comparative study (n=247) | SF36v2 and 6 question survey adapted from general facial aesthetic surgery assessment tool | Mental health and facial aesthetics |
| Alper et al., 2023 | USA | Plastic reconstructive Surgery Global Open | Cohort (n=48) | Face – Q & WHOQOL-BREF | Changes in psychological and physical quality of life and satisfaction with facial appearance, both overall and by individual features. |
| Gulati et al.,2023 | USA | Facial Plastic Surgery & Aesthetic Medicine | Observational Cross-Sectional study (n=37) | Post op Face – Q & WHaaOQOL-BREF instruments | Changes in psychological and physical quality of life and satisfaction with facial appearance. |
| Raffaini et al.,2019 | Italy | The journal of Craniofacial Surgery | Case series (n=9) | ANA Scale self evaluation post op | Satisfaction in postoperative facial aesthetic. |
|  | | | |  |  |
| **Study** | **Location** | **Journal** | **Type of study** | **Quality of life assessment tool** | **QoL Dimension** |
| Simon et al.,2022 | Spain | Plastic & Reconstructive Journal | Cohort (n=837) | 6 item questionnaires regarding pre and post op | Femininity perception |
| Capitan et al., 2014 | Spain | Plastic & Reconstructive Surgery | Observational Cross-Sectional study (n=172) | 5 item questionnaires | Surgical facial aesthetic outcome |
| Schmidt et al., 2022 | France | Oral & Maxillofacial Surgery | Cohort (n=48) | Pre and post op FACE-Q | Changes in psychological and physical quality of life |
|  |  |  |  |  | Changes in psychological and physical quality of life regarding the forehead surgery |
