# Supplementary material for: Facial Feminization Surgery and Quality of Life in Transgender Women: Protocol for a Cohort Study
Source: JMIR Res Protoc. 2025 Oct 28;14:e75065. doi: 10.2196/75065 (PMC12605289; doi:10.2196/75065)
Supplement: Multimedia Appendix 4 [file resprot_v14i1e75065_app4.docx]

**Identification of studies via other methods**

**Identification of studies via databases and registers**

Records identified from:

Websites (n =0)

Organisations (n =0)

Citation searching (n =2)

etc.

Records removed *before screening*:

Duplicate records removed (n =67 )

Records marked as ineligible by automation tools (n =0 )

Records removed for other reasons (Lenguage other than english or spanish) (n = 7 )

Records identified from*:

Databases (total = 351)

Pubmed n= 187

Scielo n=4

Google Scoolar n= 160

Registers (n =0)

**Identification**

Records screened

(n =279)

Records excluded**

(n = )

Reports not retrieved

(n =0 )

Reports sought for retrieval

(n =2)

Reports sought for retrieval

(n =105 )

Reports not retrieved

(n =174 )

**Screening**

Reports assessed for eligibility

(n =2 )

Reports excluded:

Reason 1 (n = )

Reason 2 (n = )

Reason 3 (n = )

etc.

Reports assessed for eligibility

(n =32)

Reports excluded:

Wrong study design (editor letter or review) (n =11)

Focus on surgical technique 2 (n =13)

Studies included in review

(n =10)

**Included**
