# Supplementary material for: Facial Feminization Surgery and Quality of Life in Transgender Women: Protocol for a Cohort Study
Source: JMIR Res Protoc. 2025 Oct 28;14:e75065. doi: 10.2196/75065 (PMC12605289; doi:10.2196/75065)
Supplement: Multimedia Appendix 5 [file resprot_v14i1e75065_app5.pdf]

Hospital San Juan de Dios - CDT
Asistencial Docente
"El Primero de Chile"

COMITÉ ÉTICO CIENTÍFICO
HOSPITAL SAN JUAN DE DIOS

RESEARCH PROTOCOL REVIEW CERTIFICATE

To the Editor – JMIR Research Protocols
We hope this message finds you well. We hereby inform you that the **Scientific Ethics Committee of Hospital San Juan de Dios**, re-accredited by the health authority through Exempt Resolution No. 2213578397 dated December 5, 2022, has approved the research protocol entitled:

"Multidimensional analysis of facial feminization in adult transgender women: Impact on quality of life, perception of femininity, and morphological changes",
in session No. 174, held on **July 27, 2023**.

Additionally, we inform you that the **annual renewal** of this approval was granted on **December 12, 2024**.

During the ethical evaluation process, the following documentation was submitted:
- Research Protocol (Version No. 1.0)
- Form B: Submission of scientific studies without pharmaceutical industry sponsorship
- Executive Summary
- Informed Consent Form
- Feasibility Form signed by the Department Head
- Commitment Letter from the Principal Investigator: **Francisca Donoso Hofer**
- CV of the Principal Investigator: **Francisca Donoso Hofer**
- CV of the Responsible Investigator: **Francisca Donoso Hofer**
- CVs of Co-Investigators: **Rolando Carrasco Soto, María Concepción Martín Álvaro, Marco Antonio Cornejo Ovalle**

All documents were reviewed in accordance with the principles outlined in the **Declaration of Helsinki**, the **International Ethical Guidelines for Health-Related Research Involving Humans – CIOMS 2016**, and the **ICH Guidelines for Good Clinical Practice – 1996**.

The ethical review evaluated the following aspects:

1. Scientific validity:
- Adequate human and material resources
- Appropriate methodology
- Clearly defined objectives
- Proper statistical methods, including sample size calculation

2. Social value:
- Generation of relevant knowledge
- Scientifically valuable hypothesis
- Applicability of results
- Justification even if replicating previous studies

3. Investigator competence:
- CVs demonstrating relevant experience and qualifications

4. Risk-benefit assessment:
- Minimal and foreseeable risks
- Clear procedures in case of complications

5. Fair participant selection:
- Well-defined inclusion and exclusion criteria
- No evidence of arbitrary discrimination
- Scientifically justified criteria focused on minimizing risk

6. Informed consent:
- Clear description of risks and procedures
- Forms signed by participant, investigator, and center director
- Clarity regarding voluntary participation and responsible parties

7. Protection of vulnerable populations:
- Identification of vulnerable groups and appropriate ethical procedures
- Consideration of assent when applicable

8. Privacy and confidentiality protection:
- Proper anonymization and data/sample custody mechanisms
- Defined data retention period

9. Compensation for harm:
- Declaration of mechanisms for compensation in the event of injury or harm

Conclusion:
Following a rigorous and detailed review of the submitted documentation, it has been determined that the protocol meets all ethical requirements established by national regulations as well as international standards set forth by the Declaration of Helsinki, CIOMS, and the ICH Good Clinical Practice Guidelines.
The study presents a strong scientific rationale, appropriate methodology, and a well-considered evaluation of associated risks and benefits. Additionally, it ensures the protection of participants' rights, dignity, safety, and well-being by establishing proper mechanisms for informed consent, data confidentiality, and compensation in the event of any harm.
Based on the above, the Scientific Ethics Committee has resolved to grant ethical approval for the submitted research protocol, authorizing its implementation under the evaluated terms and conditions.

The following is the list of permanent members of the Scientific Ethics Committee of Hospital San Juan de Dios:
- **José Tomás Doña**, Chair – CEC-HSJD (Lawyer)
- **Ronald Soto Arancibia**, Vice-Chair – CEC-HSJD (Physician)
- **Jimena Andrea Canales Urriola**, Executive Secretary – CEC-HSJD (Pharmaceutical Chemist)
- **Carolina Méndez Benavente**, Member – CEC-HSJD (Physician)
- **Iván Olea Silva**, Member – CEC-HSJD (Pharmaceutical Chemist)
- **Felipe Rosales Lillo**, Member – CEC-HSJD (Speech Therapist)
- **Mónica Acevedo Leyton**, Member – CEC-HSJD (Community Representative)
- **Pedro Rojas Román**, Member – CEC-HSJD (Psychologist)
- **Tamara Pulgar Vargas**, Member – CEC-HSJD (Nurse)
- **Walter Avendaño Jara**, Member – CEC-HSJD (Biomedical Engineer)

Sincerely,

HOSPITAL SAN JUAN DE DIOS
COMITÉ ÉTICO CIENTÍFICO-CEC

José Tomás Doña
Scientific Ethics Committee
Hospital San Juan de Dios
